# Apert Syndrome: Oral, Maxillofacial and Dental Management—A Narrative Clinical Review

**DOI:** 10.3390/clinpract16070135

**Published:** 2026-07-22

**Authors:** Nikolaos G. A. Kolomvos, Thomai Papadaki, Gregoris Venetis

**Affiliations:** 1 Department of Oral and Maxillofacial Surgery, School of Dentistry, National and Kapodistrian University of Athens, 2 Thivon Street, Goudi, Athens 11527, Greece; 2 Dental School, National and Kapodistrian University of Athens, 2 Thivon Street, Goudi, Athens 11527, Greece; papthomay@gmail.com; 3 Department of Oral and Maxillofacial Surgery, School of Dentistry, Aristotle University of Thessaloniki, Thessaloniki 54124, Greece

**Keywords:** FGFR2 mutation, syndromic craniosynostosis, craniofacial surgery, Le Fort III osteotomy, pseudocleft palate, dental anomalies

## Abstract

Apert syndrome is a rare genetic disorder characterized by premature fusion of the cranial sutures, syndactyly of the extremities, and distinct craniofacial deformities. The condition results from mutations in the FGFR2 gene, which disrupt normal craniofacial growth and lead to complex functional and morphological abnormalities. Patients with Apert syndrome commonly present with stomatognathic abnormalities, which significantly affect oral function and facial development. The management of Apert syndrome requires a multidisciplinary therapeutic approach. Surgical treatment strategies are typically staged according to the patient’s age and clinical severity. Early interventions focus on cranial vault expansion procedures, such as fronto-orbital advancement and posterior vault distraction osteogenesis, aiming to relieve intracranial pressure and improve cranial morphology. During childhood and adolescence, midface advancement techniques are commonly performed to address midfacial hypoplasia and associated functional impairments. Early diagnosis and appropriate surgical planning play a crucial role in preventing complications and improving the functional, aesthetic, and psychosocial outcomes of patients with Apert syndrome. This narrative review summarizes current evidence while highlighting areas of ongoing controversy, particularly regarding surgical sequencing, orthodontic management and the integration of digital technologies into multidisciplinary care.

## 1. Introduction

Apert syndrome, also known as acrocephalosyndactyly type I, is a rare genetic disorder that affects craniofacial structures and results in characteristic craniofacial deformities accompanied by syndactyly of the hands and feet. The syndrome was first described by the French physician Eugène Apert in 1906, who reported nine individuals presenting with similar clinical features [1]. Since then, it has been documented that Apert syndrome represents one of the four major types of craniosynostosis and accounts for approximately 4.5% of diagnosed cases of craniosynostosis [2]. According to the National Organization for Rare Disorders, the prevalence of the syndrome is estimated to be 1 in 65,000 live births [3]. The etiology of the syndrome is genetic, as specific mutations have been identified in the FGFR2 (Fibroblast Growth Factor Receptor-2) gene, which plays a crucial role in embryonic skeletal development. In most patients, two mutations in the FGFR2 gene have been most frequently reported in recent years, namely Ser252Trp and Pro253Arg, which are associated with distinct clinical characteristics. The Ser252Trp mutation has been reported to be associated with high rates of cleft palate occurrence, whereas the Pro253Arg mutation is more strongly associated with syndactyly [4]. These mutations usually occur de novo, as spontaneous sporadic mutations, leading to Apert syndrome even in the absence of an affected parent. However, when one of the parents is affected, the syndrome follows an autosomal dominant pattern of inheritance. While the cranial vault and limb anomalies are hallmarks of the syndrome, the profound midfacial hypoplasia and subsequent stomatognathic manifestations present significant, lifelong challenges for the patient. A defining characteristic of Apert syndrome is severe midfacial dysplasia, which not only dictates the characteristic facial appearance but also establishes a highly complex and restrictive environment for dental and occlusal development. Craniosynostosis significantly affects the normal growth and development of the skull and craniofacial structures and in most cases require surgical intervention. Craniofacial deformities consistently result in severe functional impairments, including compromised airway patency, often leading to obstructive sleep apnea, skeletal Class III malocclusion, and complex dental anomalies such as delayed eruption, severe crowding, and restricted alveolar development. The resulting skeletal open bite, narrow high-arched palate, or pseudocleft, and severe maxillary crowding require meticulous coordination between orthodontic preparation and major reconstructive procedures, such as Le Fort III midface advancement and subsequent orthognathic surgeries. Limb deformities involving the fingers and toes can severely impair simple daily activities. In addition to the fusion of digits, reduced mobility may also occur due to progressive ossification of the interphalangeal joints [5]. Consequently, the management of Apert syndrome necessitates a specialized, staged approach by Oral and Maxillofacial Surgeons and Pediatric Dentists. This narrative clinical review aims to critically summarize current concepts in the OMFS and dental management of these complex patients, focusing on surgical timing, orthodontic limitations, airway management, occlusal rehabilitation, the emerging role of digital workflows and long-term oral health maintenance, detail the collaborative OMFS and orthodontic strategies, as well as to highlight areas of controversy on surgical sequencing, orthodontic management, and the integration of digital technologies into multidisciplinary care to achieve stable, functional occlusion and improved quality of life.

## 2. Materials and Methods

The objective of this review is to provide a comprehensive clinical update on the current oral, maxillofacial, and dental challenges inherent to Apert syndrome. An extensive literature search was conducted between May and August 2025, with the final search completed in August 2025, across the electronic databases PubMed, Scopus, Web of Science, Google Scholar, and Wiley Online Library. The search strategy utilized keywords including “Apert syndrome”, “FGFR2”, “craniosynostosis”, “dental management”, “orthodontic”, “oral manifestations”, “surgical management”, “airway management”, “occlusion”, “Le Fort”, and “pediatric dentistry”. Studies published from the year 2000 onwards were considered, to ensure that the review reflects contemporary diagnostic, surgical, orthodontic, and dental management approaches relevant to current clinical practice, with a particular focus on high-impact systematic reviews, clinical practice guidelines, and significant clinical case reports published after 2010. Articles focusing on oral, dental, orthodontic, craniofacial surgical, airway and multidisciplinary management aspects of Apert syndrome were included. Priority was given to clinical guidelines, systematic reviews, major craniofacial surgical series and recent publications addressing contemporary treatment planning. Older studies were retained when they provided landmark descriptions or clinically relevant concepts that continue to inform current practice. Animal studies, conference abstracts, and publications not directly related to the objectives of the review were excluded. The retrieved evidence was qualitatively synthesized to outline the multidisciplinary management framework and the critical sequencing of interventions according to the patient’s developmental stages. Given the rarity of the condition and the heterogeneity of the available evidence, a narrative review approach was considered the most appropriate method for achieving the aims of the present study.

### 2.1. Timing and Sequencing of Interventions

The therapeutic management of patients with craniosynostosis, particularly Apert syndrome, requires a highly coordinated, staged approach to address severe midfacial hypoplasia and its associated functional impairments. Fadda and colleagues proposed a classification of therapeutic interventions divided into three main stages: from birth to two years, the developmental phase up to twelve years, and adulthood [6] (Table 1). It is important to note that early craniosynostosis management does not avoid the occurrence of malocclusion, which will require further management in the future with orthodontic treatment and possible orthognathic surgery during adolescence [7].

#### 2.1.1. Phase 1: Early Cranial Expansion

During the first months of life, procedures such as fronto-orbital advancement (FOA) or posterior vault distraction osteogenesis (PVDO) are indicated. Although primarily neurosurgical in nature, this early stage is critical for establishing the craniofacial framework upon which subsequent maxillofacial growth and occlusal development depend. In this context, early surgical intervention indirectly influences midfacial development, airway patency, and the timing of future orthodontic and surgical procedures. In most published clinical reports, FOA is typically performed between 8–12 months to protect ocular structures, restore the anterior craniofacial contour, and moderately increase intracranial volume. However, PVDO has increasingly been incorporated into modern algorithms, often performed between 3 and 16 months, as it achieves a more effective increase in intracranial volume and control of intracranial pressure [7,8,9]. The choice between FOA and PVDO is individualized according to the patient’s clinical presentation and the severity of ophthalmologic involvement.

#### 2.1.2. Phase 2: Preparation for Midface Advancement

During the developmental stage between 6 and 12 years of age, surgical strategy focuses on the management of midface hypoplasia. At this stage, subcranial midface advancement procedures are indicated, including Le Fort III osteotomy combined with distraction osteogenesis [10]. The osteotomy line extends across the superior orbital rim, the zygomatic processes, and the nasofrontal region, allowing the entire midface to be advanced anteriorly. In severe cases, monobloc frontofacial advancement may be utilized to correct marked hypertelorism, negative orbital inclination, and complex midfacial dysplasia. The age range of 6–10 years is considered a “window of opportunity” for these procedures, as the craniofacial skeleton can be mobilized more easily and effectively [11,12]. During this phase, early orthodontic intervention in patients with Apert syndrome often includes maxillary expansion using palatal appliances to address the severe transverse maxillary deficiency characteristic of the condition. This approach aims to improve arch coordination and create space for the eruption of permanent teeth, although outcomes may be limited by the underlying skeletal discrepancies associated with midfacial hypoplasia [13]. Conventional expansion devices, such as rapid maxillary expanders, may be employed; however, their effectiveness is frequently reduced in syndromic patients compared to non-syndromic cases. In addition, the presence of a pseudocleft palate introduces significant technical challenges, as the irregular palatal morphology and deep midline groove complicate both the adaptation and stabilization of the appliance. These anatomical variations may also hinder accurate impression taking, further affecting appliance fabrication and adaptation, often necessitating individualized appliance design [5]. Consequently, maxillary expansion in these patients should be regarded primarily as a preparatory step to facilitate subsequent midface advancement procedures rather than as a definitive orthodontic solution, which will be required in later stages of development during adolescence [11].

#### 2.1.3. Phase 3: Definitive Orthognathic Surgery

Following early cranial vault procedures during the first months of life and midface advancement techniques at the ages of 6–12 years old, additional corrective interventions are often required during adolescence. These may include definitive orthognathic surgery, such as Le Fort I osteotomy or bilateral sagittal split osteotomy of the mandible, aimed at achieving final occlusal correction, alongside nasofacial procedures to improve facial symmetry [7]. Le Fort I osteotomy allows for horizontal advancement, transverse widening, and vertical lengthening. Often this is combined with a genioplasty to further correct lower face deformity and occlusion. At that stage, more extensive, comprehensive orthodontic treatment, commonly referred to as presurgical orthodontic treatment, is recommended to finalize the position of the teeth and facilitate correct placement of the jaws at surgery, between the ages of 13 and 17 years old, when all permanent teeth have erupted. Extractions in the maxillary arch are frequently contemplated to relieve severe crowding and increase the surgical advancement of the maxilla. Severely impacted canines may be required to be extracted, or first premolars instead, depending on the severity of impaction and treatment plan of the case [14]. A combination of removable and fixed appliances are recommended to correct orthodontic discrepancies, as well as a transpalatal skeletal distractor and Le Fort I surgery as described above.

### 2.2. Management of “Pseudocleft” and True Cleft Palate

A narrow and high-arched palate is another common finding, sometimes accompanied by cleft palate involving both the hard and soft palate, while cleft of the uvula may also occur [15]. However, it should be emphasized that bilateral hypertrophy and enlargement of the lateral palatal processes may resemble the clinical appearance of a cleft palate, a condition referred to as pseudocleft [6,16]. These swellings are present in infancy and increase in mass as the child grows older. The cumulative tissue can proliferate to such an extent as to produce a pseudocleft in the midline, which can sometimes lead to a mistaken diagnosis of cleft palate [5]. Differentiating between a true cleft palate and a pseudocleft is essential for surgical planning and subsequent orthodontic and prosthodontic management (Table 2). In the case of a true cleft palate surgical management is required. Palatoplasty is typically performed around 11–12 months of age, aiming to optimize speech development while minimizing adverse effects on maxillofacial growth, although most repairs are carried out between 6 and 12 months. Very early intervention, earlier than 6 months of age, has been associated with higher complication and reoperation rates, whereas timing between 10 and 14 months appears to yield more favorable outcomes. It aims to restore separation between the oral and nasal cavities, enable normal feeding and speech, and reconstruct the velopharyngeal mechanism. In selected cases, such as submucous cleft palate, surgery may also be indicated when functional speech impairment becomes evident [17]. A surgical palatoplasty procedure may further compromise maxillary growth in patients affected by midfacial hypoplasia, as it occurs in patients with Apert syndrome. The pseudocleft anatomy primarily complicates orthodontic management as well as maxillary impression taking and the fabrication of orthodontic appliances, frequently necessitating customized approaches, including silicone-based impression materials to maximize accuracy and comfort of the patient while taking [18].

### 2.3. Specific Dental Anomalies and Their Management

#### Skeletal and Occlusal Disturbances

The craniofacial morphology of patients with Apert syndrome is highly characteristic. The skull typically presents acrobrachycephaly, characterized by a reduced anteroposterior cranial dimension due to the premature fusion of the coronal sutures and the absence or early closure of the sagittal or metopic sutures [19]. This premature fusion may be associated with restricted development of the brain and the central nervous system; however, a clear association with cases of reduced intellectual capacity has not been definitively established [20]. The midface region is usually hypoplastic, contributing to the clinical appearance of facial asymmetry. The maxilla is positioned posteriorly due to its deficient growth, making the mandible appear relatively prominent, resulting in pseudo-prognathism and Angle Class III malocclusion [21]. Skeletal Class III relationships can be confirmed radiographically through cephalometric analysis [16].

### 2.4. Tooth Number Abnormalities and Eruption Disturbances

Dental characteristics may vary among patients. Dental crowding, bilateral posterior crossbite and delayed eruption of both primary and permanent teeth are among the most frequently reported intraoral findings in individuals with Apert syndrome [20]. A systematic review and meta-analysis published in 2025 reported that 37% of individuals with Apert syndrome exhibit congenital absence of permanent teeth, most commonly involving the mandibular second premolars and the maxillary lateral incisors, while maxillary hypoplasia appears to be directly associated with the pattern of dental agenesis [22]. Tooth agenesis significantly compromises arch development and occlusion in patients with Apert syndrome, especially in the presence of maxillary hypoplasia and skeletal Class III relationships. Eruption of the primary dentition has also been reported to be delayed by an average of 0.96 years, while a generalized delay in dental development and formation of the dental arches has been observed during pre-adolescent and adolescent stages [19]. Ectopic eruption of permanent first molars occurs in 50% of patients with Apert, which is nearly 20 times more prevalent than in the general population. This eruption pattern may result in resorption of the distal root of the adjacent primary tooth and often create space loss that further exacerbates the already severe dental crowding. In cases where self-correction of the eruption is not achieved, intervention is required [14]. Supernumerary teeth have been described among the oral manifestations of Apert syndrome, although they occur less frequently than delayed or ectopic eruption and tooth impactions. Case reports and patient series indicate that supernumerary teeth may be present as part of the broader spectrum of dental anomalies associated with the syndrome, with reported prevalence estimated at 16.7%, highlighting its clinical importance [15,23].

### 2.5. Enamel Defects

Additionally, enamel hypoplasia is a common finding in these patients and is attributed to disturbances in the normal differentiation of odontoblasts and ameloblasts due to the altered function of the FGFR2 gene. Enamel typically presents reduced thickness, irregular porous surfaces and areas of hypomineralization, rendering teeth more susceptible to dental caries and thermal sensitivity [24]. In several cases, affecting up to 40% of patients, discolorations or white spots may also be observed, indicating enamel hypoplasia [25].

### 2.6. Impact of Syndactyly on Oral Health

Oral hygiene in patients with Apert syndrome is often compromised. This is mainly due to the restricted mobility of the upper limbs resulting from syndactyly, as well as dental crowding and ectopic eruption of teeth, factors that make effective cleaning of all tooth surfaces more difficult [19,26]. In a study investigating oral health and masticatory ability in patients with different types of craniosynostosis, a significantly higher risk of dental caries and poorer oral hygiene was observed compared with the control group. Valid indices such as the Simplified Oral Hygiene Index (OHI-S) were used to evaluate dental plaque accumulation and calculus deposition on tooth surfaces. Additional factors contributing to compromised oral health included neurological impairments, reduced visual acuity, decreased tongue mobility, and reduced salivary flow, all of which further complicate the maintenance of adequate oral hygiene in these patients [15]. In another study conducted by Dalben et al., dental plaque accumulation, calculus formation, and caries prevalence were investigated in patients with syndromic craniosynostosis, ten of whom had been diagnosed with Apert syndrome. The indices Personal Hygiene Performance (PHP), assessed before and after unsupervised tooth brushing, as well as DMFT indices (Decayed, Missing, Filled Teeth) for primary and permanent dentition respectively, were used for evaluation. The results demonstrated high levels of dental plaque, mainly due to reduced brushing ability, although gingival inflammation levels were not significantly elevated. Additionally, 42.1% of the sample required restorative treatment for carious lesions [27]. According to a study by Mufalo et al., an increased risk of periodontal disease at a young age in patients with craniosynostosis has not yet been clearly established. Nevertheless, increased periodontal pocket depth in posterior teeth and greater attachment loss have been observed, highlighting the importance of regular periodontal monitoring and professional scaling procedures every three months, as well as use of fluoridated toothpaste for plaque control and removal twice a day [26,28]. New generation electric toothbrushes are also recommended in Apert syndrome patients due to the ergonomic handles and newly developed technologies for controlling the time of daily brushing and applied pressure [29]. The use of customized toothbrush handles has been described as a practical adaptation to enhance grip and improve plaque control in daily oral hygiene routines. These types of handles may be fabricated using silicone-based impression materials by taking an impression of the patient’s hand or grasping pattern and incorporating it into the handle design. This individualized approach enhances grip and control, facilitating more effective oral hygiene in patients with limited manual dexterity due to syndactyly [26].

### 2.7. Airway Management and Anesthesia Consideration in Dental Chair

The craniofacial deformities inherent to Apert syndrome, such as midface hypoplasia, reduced upper airway dimensions, consistently result in severe functional respiratory impairments. Nasal airway obstruction, chronic oral breathing, and obstructive sleep apnea are frequently observed and are largely attributed to the concave facial profile and the reduced volume of the nasopharyngeal and oropharyngeal spaces resulting from midfacial hypoplasia. Screening for sleep-disordered breathing and obstructive sleep apnea should be considered prior to procedures requiring sedation or general anesthesia. These respiratory challenges require continuous evaluation of airway function by the multidisciplinary team [30]. Preoperative airway evaluation is critical, as well as anesthetic planning must account for these challenges. In the context of dental and OMFS management, while routine preventive and minimally invasive dental procedures can often be performed safely in the outpatient setting, patients requiring extensive dental or surgical procedures with prolonged treatment sessions, deep sedation or presenting with significant airway compromise may benefit from management in a hospital-based environment. Preoperative airway evaluation is critical, and anesthetic planning must account for these challenges. In such cases, access to specialized anesthetic support and multidisciplinary care may reduce perioperative risks and facilitate the safe delivery of dental treatment, while ensuring airway safety [31].

## 3. Discussion

The craniofacial characteristics described above represent direct manifestations of the pathological activation of the FGFR2 receptor and are closely associated with the severity of the clinical presentation of Apert syndrome as well as with its therapeutic requirements. Taken together, the available evidence highlights that successful management of Apert syndrome extends beyond correction of craniosynostosis and requires long-term coordination of craniofacial, orthodontic, and dental interventions. Within this context, the diagnosis of Apert syndrome emerges as a crucial factor not only for identifying the phenotypic characteristics of the disorder but primarily for enabling the timely planning and coordination of multidisciplinary management. Early diagnosis is directly associated with parental counseling and decision-making regarding pregnancy, particularly in severe forms of the syndrome. Traditionally, the diagnosis of Apert syndrome has been based on the combination of characteristic cranial morphology and complex syndactyly. However, the recent literature indicates that early clinical recognition is not always feasible before the third trimester of pregnancy, as craniofacial abnormalities often become more apparent during later stages of gestation. The contemporary diagnostic approach to Apert syndrome relies on a combination of clinical, imaging, and genetic evaluation. Genetic confirmation through the detection of mutations in the FGFR2 gene has gained increasing importance in recent years, not only for establishing a definitive diagnosis but also for differentiating Apert syndrome from other syndromic craniosynostoses with overlapping phenotypic characteristics, such as Crouzon or Pfeiffer syndrome. Definitive confirmation of the diagnosis is achieved through molecular genetic analysis of the FGFR2 gene. According to recent studies, more than 98% of Apert syndrome cases are attributed to two recurrent mutations in the FGFR2 gene, namely Ser252Trp and Pro253Arg, which alter the function of the fibroblast growth factor receptor and lead to premature ossification of the cranial sutures. Furthermore, the association between specific mutations and the extent of syndactyly or the presence of cleft palate provides prognostic value and supports the development of personalized treatment strategies [32]. Syndromic craniosynostosis is considerably more complex than isolated non-syndromic craniosynostosis, as it often requires multiple surgical interventions and is associated with more severe clinical manifestations and greater patient burden. The possibility of early diagnosis of Apert syndrome allows the timely initiation of appropriate therapeutic strategies and optimal timing of surgical interventions. The clinical significance of early diagnosis extends beyond the recognition of the syndrome itself, as it also contributes to the prevention of potential functional complications and improvement of patients’ overall quality of life. Multiple surgical procedures beginning in early infancy, including cranial vault reconstruction and midface advancement surgeries, may significantly affect the psychosocial and physical well-being of patients. Despite the established role of Le Fort III advancement in the management of severe midface hypoplasia during childhood, the optimal timing of intervention remains controversial. The controversy regarding the timing of midface advancement reflects a balance between functional urgency and long-term skeletal stability. Earlier intervention may be justified in the presence of airway compromise, ocular exposure, or severe psychosocial burden, but it may also increase the likelihood of secondary procedures because craniofacial growth is incomplete. Conversely, delaying definitive advancement may improve skeletal predictability but can prolong functional impairment and facial imbalance during childhood. Therefore, the current literature does not support a universal timing protocol; rather, treatment should be individualized according to airway status, ocular protection, occlusal development, psychosocial needs, and center-specific expertise. It appears to be often individualized depending on several factors such as airway compromise, ocular protection, psychosocial condition of the patient, and craniofacial growth considerations. Although distraction-based approaches have gained widespread acceptance because they allow greater advancement with improved soft-tissue adaptation, high-quality comparative evidence remains limited, and no universal consensus has been established regarding the optimal surgical protocol. Additionally, further orthognathic procedures are often required later in life to correct skeletal discrepancies, further increasing the physical and psychological burden on affected individuals [19]. Evidence from the literature regarding oral health in patients with Apert syndrome indicates increased levels of dental plaque accumulation and gingivitis compared with the general population. This tendency is mainly attributed to the dental and skeletal abnormalities associated with the syndrome, which hinder effective oral hygiene practices. Orthodontic and dental anomalies commonly observed in Apert syndrome, such as severe dental crowding, ectopic tooth eruption, and skeletal Class III malocclusion, significantly affect mastication, speech, and the maintenance of adequate oral hygiene, as reported by Carpentier et al. [33]. Furthermore, syndactyly of the upper limbs and reduced finger mobility may impair patients’ ability to maintain adequate oral hygiene independently, contributing to the accumulation of dental plaque. These findings strongly suggest that patients with syndromic craniosynostosis, including Apert syndrome, often face significant challenges in maintaining proper oral hygiene due to limitations in manual dexterity associated with syndactyly. Despite the increased levels of plaque accumulation, risk of gingival inflammation and caries, no standardized preventive dental protocol specifically designed for Apert syndrome currently exists. Alongside traditional multidisciplinary approaches, emerging digital technologies may further enhance the management of patients with Apert syndrome. Intraoral and facial scanning provide non-invasive, radiation free techniques for documenting complex craniofacial and dental deformities, alleviating the challenges linked to conventional impression procedures, particularly in patients presenting with severe maxillary constriction or palatal pseudoclefts. Digital workflows have recently demonstrated significant clinical value in the management of other neonatal craniofacial anomalies as well. Čverha et al. described a fully digital workflow combining intraoral scanning, CAD design and three-dimensional printing for the fabrication of wire-free palato-lingual plates in infants with Robin sequence, eliminating the need for conventional impressions and extraoral retention wires while improving patient comfort and manufacturing efficiency. This contemporary application highlights the expanding role of additive manufacturing and digital technologies in infant craniofacial care and supports their potential integration into the management of complex craniofacial conditions, including Apert syndrome [34]. In addition, digital workflows incorporating three-dimensional imaging, virtual surgical planning, and CAD/CAM technologies may improve the accuracy of orthodontic treatment planning and craniofacial surgical interventions [35]. Virtual Surgical Planning (VSP) and CAD/CAM-assisted workflows enable the three-dimensional modeling of osteotomies, distraction vectors and postoperative skeletal relationships alignments prior to the operation [36]. In patients with Apert syndrome, these technologies may aid in the planning of midface advancement surgery and distraction osteogenesis by allowing precise evaluation of skeletal anatomy and structures and customization of surgical guides. Recent research has demonstrated that patient-specific cutting guides enhance the accuracy and safety of complex osteotomies procedures, particularly in cases involving severe midfacial hypoplasia and atypical craniofacial anatomy. Furthermore, by minimizing intraoperative trial-and-error and improving preoperative decision-making, VSP may lead to shorter operative and anesthesia times, more predictable and reliable outcomes, as well as reduced perioperative morbidity [37]. However, most available evidence regarding VSP in syndromic craniosynostosis derives from case reports, technical notes, and small surgical series. Therefore, although these technologies appear promising, their impact on long-term stability, complication rates, cost-effectiveness, and patient-reported outcomes remains insufficiently established. As experience with these technologies continues to expand, digital workflows are progressively evolving from adjunctive, diagnostic tools to integral components of contemporary craniofacial surgical practice. The integration of facial scans with digital dental records enables thorough evaluation of occlusal relationships and facial symmetry, enhancing individualized treatment strategies and interdisciplinary communication [38]. In this context, management of Apert syndrome requires the close collaboration of multiple medical specialties and the establishment of a multidisciplinary team, including oral and maxillofacial surgeons, orthodontists, pediatric dentists, otorhinolaryngologists, speech therapists, and occupational therapists, aiming to restore functional capacity and improve the daily life of affected individuals. Importance should also be attributed to the role of the general dentist, who contributes both to the early recognition of stomatognathic manifestations and to the maintenance of oral health through regular preventive examinations and appropriate therapeutic interventions when necessary.

## 4. Limitations

Several limitations of the present review should be acknowledged. This research was structured as a narrative review and consequently does not adhere to the methodological framework typical of a systematic review. As a result, no formal study quality assessment, risk-of-bias evaluation, or quantitative synthesis was performed. Furthermore, the available literature on Apert syndrome remains inherently limited due to the rarity of the condition, with a significant portion of the published evidence consisting mainly of case reports, retrospective case series, and specialized centers insights. In addition, considerable heterogeneity exists among published studies concerning treatment protocols, timing of surgical interventions, orthodontic strategies, and outcome assessment. Therefore, the clinical recommendations discussed in this review should be interpreted within the context of the currently available evidence. Nevertheless, the present review aims to deliver a clinically relevant synthesis of contemporary concepts in the oral, maxillofacial, and dental care of patients with Apert syndrome.

## 5. Conclusions

The management of Apert syndrome presents a highly complex set of craniofacial, stomatognathic, and dental challenges that necessitate a well-coordinated, multidisciplinary approach. As highlighted in this review, therapeutic interventions must be carefully staged, from early cranial vault expansion and midface advancement to definitive orthognathic surgery, to align seamlessly with the patient’s skeletal growth and dental development. Current evidence supports early cranial vault interventions such as fronto-orbital advancement (FOA) and posterior vault distraction osteogenesis (PVDO), within the first 24 months of life, while midface advancement surgeries are typically regarded during childhood, especially between 6 and 10 years, based on airway and ocular assessment as well as psychosocial factors. Definitive orthognathic surgeries such as Le Fort I osteotomies are typically reserved for skeletal maturity. However, significant challenges persist, including inconsistency in treatment protocols across specialized craniofacial centers, ambiguity surrounding the ideal timing for midface advancement procedures and the absence of syndrome-specific preventive dental protocols. Oral and maxillofacial surgeons, pediatric dentists, and orthodontists are essential not only in achieving functional occlusal rehabilitation but also for addressing syndrome-specific anomalies such as palatal pseudoclefts, severe crowding, and ectopic eruptions. The distinction between true cleft palate and pseudocleft palate is of critical importance clinically, as it can directly impact surgical, orthodontic interventions and the practicality of individualized maxillary expansion methods and treatment planning. Furthermore, customized preventive strategies are essential to overcome the oral hygiene limitations imposed by syndactyly, as well as rigorous preoperative airway assessment for safe anesthetic management before any procedure that necessitate sedation or general anesthesia. Digital workflows that include intraoral scanning, facial scanning, CAD/CAM technologies and Virtual Surgical Planning (VSP) are progressively changing modern craniofacial practice. In addition to enhancing diagnosis and treatment strategies, these technologies enable precise surgical performance, optimization of distraction osteogenesis and a decrease in operative morbidity. As their application keeps growing, VSP is gradually evolving from a developing technology to a fundamental aspect of contemporary craniofacial surgical treatment. Ultimately, early intervention, individualized surgical planning, and continuous, specialized dental care are paramount to optimizing both the functional outcomes and the overall quality of life for patients with Apert syndrome.

Keypoints:

1. Apert syndrome is a rare genetic disorder characterized by premature fusion of the cranial sutures and distinct craniofacial and stomatognathic abnormalities.
2. Patients frequently present maxillary hypoplasia, severe occlusal disturbances and pronounced dental crowding, which may significantly affect oral health and the functional performance of the stomatognathic system.
3. Early diagnosis is crucial for preventing complications such as increased intracranial pressure and for the appropriate planning of therapeutic interventions.
4. Management of these patients requires close collaboration among a multidisciplinary team, including oral and maxillofacial surgeons, orthodontists, pediatric dentists, speech and occupation therapists as well as other healthcare professionals.
5. Regular dental monitoring and early orthodontic intervention may significantly contribute to improving both functional outcomes and the overall quality of life of patients with Apert syndrome.
6. Digital workflows, including intraoral scanning, facial scanning, CAD/CAM technologies, and VSP, are increasingly being incorporated into specialized craniofacial centers. These tools may assist in documentation, interdisciplinary communication, surgical simulation, and guide fabrication. However, their role should currently be interpreted as adjunctive, since comparative evidence regarding long-term outcomes in Apert syndrome remains limited.

## Figures and Tables

**Table 1:** Multidisciplinary Team Management Timeline for Apert Syndrome.

Age	Craniofacial Priorities	Dental and Orthodontic Priorities	Surgical/Medical Priorities
**Birth–2 years**	Craniofacial assessment, ICP monitoring, airway evaluation	Oral hygiene and nutritional guidance, feeding support, eruption monitoring	FOA/PVDO, syndactyly release, ophthalmologic and hearing assessment
**3–5 years**	Monitoring craniofacial growth	Preventive dental care	Speech evaluation, airway monitoring
**6–10 years**	Management of midface hypoplasia	Maxillary expansion, Phase I Orthodontics	Le Fort III distraction osteogenesis (when indicated), Monobloc advancement (selected cases)
**10–12 years**	Post-surgical craniofacial follow up	Orthodontic retention, eruption monitoring	Secondary corrections (if required)
**13–18 years**	Final skeletal assessment and facial balance	Comprehensive orthodontic treatment, Phase II Orthodontics	Orthognathic surgery, Le Fort I osteotomy, BSSO, rhinoplasty, facial symmetry and soft tissue/esthetic corrections
**Adulthood**	Long-term follow up	Periodontal maintenance, prosthetic and preventive care	Secondary revisions (if required)

**Table 2:** Differentiating between true cleft palate and “pseudoclet” palate in Apert Syndrome.

Feature	True Cleft Palate	Pseudocleft Palate
**Anatomical defects**	True discontinuity of the palatal tissues with communication between oral and nasal cavity	Midline cleft resulting from bilateral hypertrophy of lateral palatal shelves, lacking actual tissue separation
**Surgical considerations**	Palatoplasty in infancy	No surgical intervention is typically required
**Orthodontic considerations**	Influences maxillary growth and necessitates multidisciplinary care	Complicates appliance adaptation and maxillary expansion due to atypical palatal morphology
**Speech and feeding**	Associated with feeding compromising and velopharyngeal dysfunction	Less severe functional impairment
**Impression taking and appliance fabrication**	Conventional cleft palate protocols to be followed	Complicates accurate impression taking and appliance fabrication
**Clinical implication**	Requires surgical assessment and usually palatoplasty before comprehensive orthodontic planning	Does not usually require surgical closure but may complicate impressions, appliance design, and maxillary expansion

## Data Availability

No new data were created or analyzed in this study. Data sharing is not applicable to this article.
